# Central Effects of Camphor on GnRH and Sexual Hormones in Male Rat

**Published:** 2012

**Authors:** Sima Shahabi, Seyed Gholam Ali Jorsaraei, Ali Akbar Moghadamnia, Ebrahim Zabihi, Seyed Mohsen Aghajanpour, Seyedeh Narges Mousavi Kani, Roghieh Pourbagher, Seyed Ahmad Hosseini, Mohsen Esmaili, Ali Asghar Yoonesi, Amin Zarghami, Farid Alinezhad

**Affiliations:** 1 *Cellular and Molecular Biology Research Center (CMBRC), Babol University of Medical Sciences, Babol, Iran.*; 2 *Fatemeh Zahra Fertility and Infertility Reproductive Health Research Center, Babol University of Medical Sciences, Babol, Iran.*; 3 *Department of Physiology and Pharmacology, Babol University of Medical Sciences, Babol, Iran.*; 4 *Student Research Committee, Babol University of Medical Sciences, Babol, Iran.*

**Keywords:** Camphor, GnRH, Hypothalamus - Pituitary - Gonad (H-P-G) Axis

## Abstract

In Persian traditional medicine is believed that camphor (a crystalline ketone obtained from cinnamomum camphora) is a suppressor of sexual behaviors. This study examined the central effects of camphor on sexual hormones (LH, FSH and testosterone) and GnRH plasma levels in male rat.

Male Wistar rats weighing 250-260gr were selected and divided into control (no treatment), sham (ICV injection of EtOH 10%) and treatment (ICV injection of camphor in three doses 4, 20, 40 µg/ 10µl in alcohol) groups. The serum samples were used for assaying of GnRH, LH, FSH and testosterone.

There were no significant differences in the levels of hormones between the groups of study.

Despite the central administration of camphor in hypothalamus - pituitary - gonad (HPG) axis, no significant differences were seen in sex hormone`s levels compared to the control. With this finding, it can be concluded that camphor may not effectively handle the axis via central pathway. These data recommend further studies of camphor on the HPG axis.

Camphor (C_10_H_16O_) is a waxy, white crystalline substance or transparent with a solid form and a strong aromatic odor. It is a ketone body from camphor laurel wood (*cinnamomum camphora*), a large evergreen tree found in Asia (1-3). It can also be synthetically produced from oil of turpentine. Solubility of camphor is 1 g about 800 ml water, 1 ml alcohol, 0.5 ml chloroform and 1 ml ether, freely soluble in carbon disulfide, and volatile oils (3). Its ways of entering the body are through food, skin, eye contact and breathing (4). The symptoms of oral camphor poisoning have been reported as blurred vision, nausea, vomiting, colitis, dizziness, delirium, contraction of heart muscles, and difficulty in breathing, seizures and death. In large quantities, it is poisonous when ingested and can cause seizures, confusion, irritability and neuromuscular hyperactivity. Lethal doses in adults are in range 50- 500 mg/kg (orally). Generally 2 g causes serious toxicity and 4 g is potentially lethal. It was also reported as an irritant of the sexual organs (5-7).

Camphor can be absorbed from skin, GI (5-90 min after ingestion) and respiratory system. It is conjugated in the liver with glucuronic acid to become soluble in water. Camphor crosses the placental barrier it may be account for its embryo toxic effects (5).

Camphor has various applications in different countries especially the Asian countries. As a form of herbal medicine, it serves as antiseptic, anti-cold (8, 9), a synthetic drug in the form of ointments, lotion and gel for anti-insect bites, antimicrobial, embalming, firework, U.V filter cream, topical analgesic, anti-itching, skin cooling, anti-burns and sunburn (10). Also, it is used in plastic retrofitting against solar radiation, pesticides, shining materials, toilet products preservatives, cosmetics, religious ceremonies chewing gum and cigarette (8-10).

Camphor had been administered in cumulative doses to induce convulsion attacks in psychiatric patients (11).

Recent studies showed that organic compound such as camphor reduce cytochrome P_450_ B_1_ activity. This enzyme interferes with one of the key enzymes in the testosterone synthesis called 17-α hydroxilase. By reducing cytochrome, the mentioned enzymes function reduces, so testosterone diminishes (12-15). Some researchers investigated the impact of camphor in UV - filter ointment on gonadotropins and gonadal hormones and concluded that camphor causes adolescent retardation and reduction in reproductive organs volumes in both sexes (16, 17). It has been shown in another study that the use of ointments containing camphor did not affect gonadotropins (LH and FSH) and testosterone (18) and it has also been demonstrated that UV - filters ointment inhibit 17 b - hydroxysteroid dehydrogenase type 3 which catalyzes the last step of testosterone synthesis in testicular leydig cells and plays an essential role during male sexual development (19).

Also, an adverse effect of camphor on rats causing a slight reduction of GnRH, LH and FSH (only in a low dose) was observed in an in vitro study (20). The effect of camphor on estrogenic gene expression (21) and estrogen receptor activity (22) was also studied.

According to Avecina's medicine, camphor is a suppressor of libido and acts as an effective element in reproductive system (23).

In Iran's folk medicine, camphor has been used as a suppressor of sexual activity. This study examined the effects of ICV (Intracerebroventricular) injection of camphor on LH, FSH, testosterone and GnRH hormones in male rat. This study was approved by the Ethic Committee of Babol University of Medical Sciences (Babol, Iran). Male Wistar rats weighing 250 - 260 gr were used. The rats were kept under standard living regimens (12hrs lights on and 12hrs lights off) and access to rat chow and water *at libetum* (free feeding means free access to chow and water thereby allowing the animal to self - regulate intake according to its biological needs during the experiments).

**Drugs and biochemical reagents**

Camphor was obtained from FREY+LAU Co. (Germany) and Ethanol, Diethyl ether, Formalin, ketamine from Merck Co. (Germany). Acrylic dental cement with solvent was purchased from Vertex Co. and Enzyme-linked immuno-sorbent assay (ELISA) Kit of Rat LH, FSH and Testosterone from USCN Life Science and Technology Company. LH-RH (GnRH) Kit of rat was purchased from Phoenix Pharmaceuticals Inc.

**Surgical procedure**

The rats were anesthetized with ketamine (75 mg/kg). For ICV injection, the rats were implanted with unilateral guide cannula (21 gauges). The stereotaxic coordinates for the cannulation into the lateral ventricle were as follows: AP -0.8 mm (from bregma), ML = 1.5 mm lateral (from midline), and DV = -3.5 mm ventral (from the surface of the skull). The incisor bar on the stereotax was set to 0.0 mm above the intraoral line (24). All animals were single housed following surgery, and were allowed to recover for 5 days before microinjections. Two stainless steel anchoring screws were fixed to the skull, and the cannula was secured in place by acrylic dental cement. The animals were allowed to recover for at least 5 days and were handled every day before the start of the experiments.

**ICV injections**

After 5 days of the establishment of ICV. cannulation, 10 μl of vehicle and camphor + vehicle were injected using a 10 μl Hamilton syringe connected polyethylene tube with a 29-gauge to internal cannula. Fifty six male rats were selected and divided into control, sham and treatment groups. The control group did not receive any materials. Sham group, received ICV injection of the vehicle (Ethanol 10 %) and treatment groups received ICV injection of the camphor (4µg, 20µg, 40µg) per 10 µl solution. 10% alcohol was used as solvent, because it was one of the best solvents for camphor. The low dilution rate 10% was not a matter of concern. In many reports, alcohol dilution further than 15% affected the reproductive system (25). ICV injection was performed through the cannula implanted on rat brain by the stereotaxic instrument, according to Paxinose Atlas with Hamilton syringe. The blood samples were collected from all animals by orbital sinus blood collection technique (26, 27) and were centrifuged and freezed at-20c. Sera were used for GnRH, LH, FSH and testosterone measurement by rat specific ELISA kit.

**Statistical Analysis**

Data were analyzed using one way ANOVA followed by Tykey post-hoc test. The difference between data in each point was considered significant at p <0.05.

## Results

Serum GnRH and LH levels did not show any significant differences in camphor receiving rats in test group when compared with the control and sham groups.

FSH was decreased by the dose of 4 µg/ 10 µl ethanol and testosterone was increased by the dose of 4 µg/ 10 µl & 40 µg/ 10 µl ethanol. Although some differences were observed, but these differences were not significant (Table 1).

**Table1:** GnRH, LH, FSH and testosterone concentration in adult male rats after receiving ICV injections of camphor (4, 20, 40 µg/10 µl ethanol 10 %) v.s control and sham groups

Group		GnRH(Mean± SD) (ng/ml)	LH (Mean± SD) (mIU/mL)	FSH (Mean± SD) (mIU/mL)	Testosterone(Mean± SD) (nmol/L)
Control		0.15±0.35	29.76 ±18.62	2.84 ±0.07	1.01± 0.75
Sham		0.31± 0.00	29.13 ±15.80	1.09 ± 0.35	0.69 ± 0.50
	4µg/10µl	0.45±0.07	32.41± 24.75	0.84 ± 0.01	1.69 ± 3.90
Test	20µg/10µl	0.30±0.14	38.24 ± 39.60	1.27 ± 0.80	1.54 ± 0.00
	40µg/10µl	0.34± 0.42	43.18 ± 8.27	1.70 ± 2.05	4.48 ± 0.56

Values in table are presented with Mean ± SD; p <0.05

## Discussion

As yet, the different results of camphor effects on the reproductive system were reported by several researchers (12-19). In Iranian folk medicine, camphor is a suppressor of sexual activity (23). In the present study we analyzed the GnRH, LH, FSH and testosterone plasma concentrations of male rats by ICV injections in various concentration of camphor. ICV injections could cause this effect, because drugs and materials can be transported in brain such as H-P-G axis via the cerebrospinal fluid of intracerebroventricular (28, 29).

GnRH and gonadotropins (LH, FSH) are a key regulator of neuroendocrine system in mammals. It is well known that the hypothalamic GnRH and hypophysis gonadotropins elicit spermatogenesis and sexual behavior by hypothalamic – pituitary – gonad (H-P-G) axis currency. Gonadal hormones such as testosterone in males have a negative feedback effect on the anterior pituitary and hypothalamus. This hormone has a strong direct effect on the anterior pituitary gland by inhibiting the secretion of gonadotropins and probably a direct effect on hypothalamus by decreasing the secretion of GnRH. Thus, whenever secretion of testosterone becomes too great, this automatic negative feedback effect, operating through the hypothalamus and anterior pituitary gland, reduces the testosterone secretion back toward the desired operating level and conversely too little testosterone allows the hypothalamus to secret large amount of GnRH corresponding to an increase in anterior pituitary LH and FSH secretion and a consequent increase in testicular testosterone secretion (30). 

In this study, the effect of ICV injection of camphor on GnRH, LH, FSH and testosterone concentrations showed no significant changes of GnRH and sexual hormones. GnRH induces spermatogenesis and sexual behavior by Hypothalamic – Pituitary – Gonad (H-P-G) axis. The effect of camphor on hypothalamic – pituitary – gonad (H-P-G) axis could be shown by ICV injection of camphor in guide canula.

Carou et al. 2008, investigated the effects of substances contained in camphor on male rats in vitro and found a reduction of GnRH (20). These results might be due to the use of camphor derivatives instead of pure camphor and in vitro environment. But in the present experiment we used pure camphor through ICV injections.

The LH levels remained unchanged despite testosterone increase. Although the differences have been shown on testosterone concentration, but these differences are not significant and are independent of GnRH and LH levels. Our results can indicate that camphor does not exerce its effect through H-P-G axis. Our results also showed FSH decrease in one group. This reduction can be a direct effect (31) in this dose of camphor on FSH secretion but is not significant. There are some studies in contradiction with our findings. Some other researchers found that camphor component in UV filter ointment can interact with gonadotropins and gonadal hormones and concluded that camphor causes adolescence retardation and reduction of reproductive organs volumes in both sexes (16).

Also it was shown in another study that the use of ointments containing camphor did not affect gonadotropins (LH, FSH) and induces little changes of testosterone (18). In an other study, subcutaneous injection of camphor containing compound during 5 days on male rats causesed slight GnRH, LH and FSH reduction (only in a low dose) as well (20). It is noteworthy that they used products containing camphor while we applied pure camphor. Thus, this difference may explain such contraversy in our results.

It can be concluded that the microinjection of pure camphor dissolved in 10% alcohol cannot affect the level of GnRH and sexual hormones. These data reinforce the need for further study of the camphor effect on hypothalamus - pituitary - gonad (H-P-G) Axis.
